# Targeting constitutive NF-κB specifically in tumor cells

**DOI:** 10.18632/oncotarget.22069

**Published:** 2017-10-25

**Authors:** Ji-Hak Jeong, Shohreh Iravani Dickinson, Jun-Li Luo

**Affiliations:** Jun-Li Luo: Department of Molecular Medicine, The Scripps Research Institute, Jupiter, FL, USA

**Keywords:** constitutive NF-κB, prostate cancer, tumor recurrence, therapy-resistance, constitutive signaling circuit

NF-κB transcription factors are the key regulators of innate and adaptive immune responses, inflammation, and cell survival [1]. Considerable evidences indicate that NF-κB plays important roles in carcinogenesis, cancer progression, metastasis and drug resistance, and therefore NF-κB has been regarded as one of the most important targets for cancer therapy [2]. However, the application of inhibitors that target NF-κB or NF-κB pathways for the treatment of human cancer is impeded by severe side effects due to the indiscriminate inhibition of NF-κB in normal immune cells.

Traditionally, there are two NF-κB activation pathways that play important roles in immune responses. The first pathway, also called canonical or classical NF-κB activation pathway, is triggered in response to microbial and viral infections and exposure to proinflammatory cytokines that induce activation of the tripartite IKK complex and the consequent phosphorylation-related degradation of IκBs, leading to the nuclear translocation of NF-κB complexes, predominantly p50/RelA and p50/c-Rel dimmers [3]. The second pathway, also called non-canonical or alternative NF-κB activation pathway, involves different signaling molecules and leads to the predominant activation of p52:RelB NF-κB dimers by inducing processing NF-κB2/p100 precursor that binds to the RelB NF-κB subunit in the cytoplasm [4].

As more and more reports suggest that NF-κB is activated in most cancer cell lines and tumor cells, especially in those tumor cells that resist to chemo- or/and radiotherapy, it becomes a consensus for the research community that NF-κB plays a major role in tumor development and cancer therapy-resistance. However, we seemed ignore the differences between NF-κB (acute) activation in immune responses and NF-κB constitutive activation in tumor cells, and also ignore or sometimes confuse the differences between the activation of NF-κB pathways and the activation of NF-κB itself. In fact, as demonstrated in both transgenic/knockout and allograft/xenograf mouse models in our studies [5, 6], the constitutive activation of NF-κB in tumor cells is different from NF-κB (acute) activation in immune cells, and the constitutive activation of NF-κB in tumor cells doesn’t dependent on traditional NF-κB pathways, which are normally related to the activation of IKKβ or IKK complex [5]. This can explain why the IKKβ/IKK inhibitors are not as powerful for the inhibition of tumor cell proliferation and tumor growth as expected or as what theoretically they should do.

**Figure 1:**
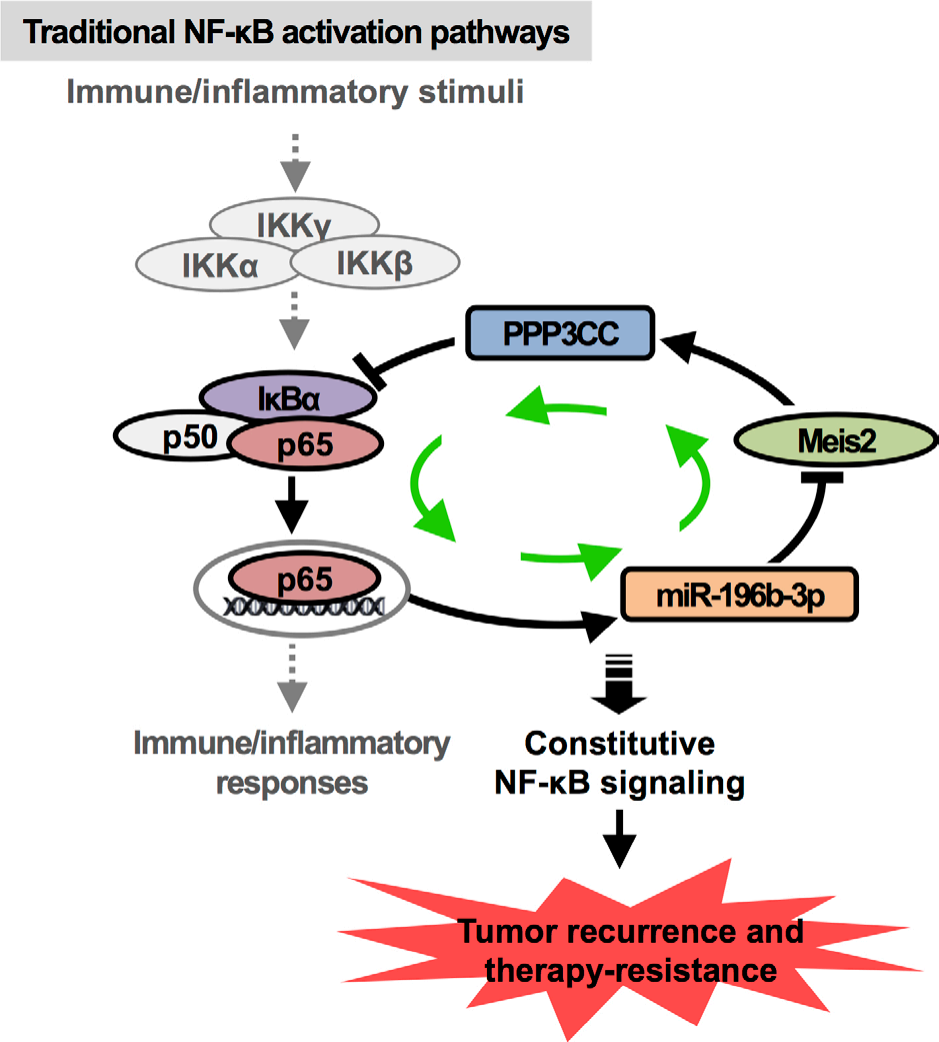
Diagram of constitutively activated NF-κB signaling circuit in recurrent or therapy-resistant prostate tumor Constitutively activated NF-κB signaling, maintained by a feed-forward circuit in castration-resistant prostate cancer, is not dependent on traditional IKKβ/NF-κB pathways.

Importantly, our studies have shown that it is the constitutive NF-κB activation that drives tumor prostate cancer recurrence and therapy-resistance. And the constitutive NF-κB activation is established and maintained by a feed-forward signaling circuit composed of IκBα/NF-κB(p65), miR-196b-3p, Meis2, PPP3CC, where the constitutive IκBα/NF-κB(p65) drives the expression of miR-196b-3p that inhibits the expression of Meis2 and PPP3CC, down-regulated PPP3CC is unable to dephosphorylates p-IκBα, leading to constitutive IκBα phosphorylation and NF-κB(p65) activation (Figure 1). Therefore, targeting this constitutive NF-κB signaling circuit by interrupting its non-IκBα/NF-κB(p65) components would be a highly efficient way for the treatment of recurrent and therapy-resistant prostate cancer, which will avoid the side effects related to indiscriminate IKK/NF-κB inhibition in normal cells.

The constitutive NF-κB signaling circuit controls the expression of a group of stem cell transcription factors, including Twist2, Sox2, Oct4, and Nanog, that drives the proliferation and self-renewal of cancer stem cells, leading to tumor recurrence and cancer therapy-resistance [5]. Although these stem cell transcription factors may not be the downstream targets of the individual non-IκBα/NF-κB(p65) components of the circuit, disrupting this circuit by targeting any of the non-IκBα/NF-κB(p65) individual components blocks the expression of these transcription factors and significantly impairs castration-resistant prostate cancer (CRPC) development [5]. Therefore, disrupting this constitutive NF-κB signaling circuit by targeting any of its individual non-IκBα/NF-κB(p65) components would be as powerful as direct inhibition of NF-κB for the treatment of CRPC.

The non-IκBα/NF-κB(p65) components in constitutive NF-κB signaling circuit include miR-196b-3p, Meis2, and PPP3CC (Protein Phosphatase 3, Catalytic Subunit, Gamma Isozyme). Further studies are needed to distinguish the best suitable one among these three non-IκBα/NF-κB(p65) components for targeting constitutive NF-κB signaling in tumor cells. It should be mentioned that the phenomenon and the role of constitutive NF-κB signaling in tumor recurrence and therapy-resistance were found in CRPC cells, further studies are needed to define if the constitutive NF-κB signaling circuit also exists in other types of recurrent and therapy-resistant cancer cells. We predict that the constitutive NF-κB signaling may be a common phenomenon in most recurrent and therapy-resistant cancer cells, however, the components of the constitutive NF-κB signaling may be different in different types of tumors, as evidenced in our previous studies on breast cancer [7].
